# Layer-specific strain analysis in patients with suspected stable angina pectoris and apparently normal left ventricular wall motion

**DOI:** 10.1186/s12947-018-0144-9

**Published:** 2018-09-26

**Authors:** Mustafa Adem Yılmaztepe, Fatih Mehmet Uçar

**Affiliations:** Department of Cardiology, School of Medicine, Trakya University, 22030 Edirne, Turkey

**Keywords:** Coronary artery disease, Left ventricle function, Echocardiography, 2D speckle tracking, Layer specific strain

## Abstract

**Background:**

Non-invasive imaging tests are widely used in the evaluation of stable angina pectoris (SAP). Despite these tests, non-significant coronary lesions are not a rare finding in patients undergoing elective coronary angiography (CAG). Two-dimensional (2D) speckle tracking global longitudinal strain (GLS) imaging is a more sensitive and accurate technique for measuring LV function than conventional 2D methods. Layer-specific strain analysis is a relatively new method that provides endocardial and epicardial myocardial layer assessment. The aim of the present study was to evaluate longitudinal layer-specific strain (LSS) imaging in patients with suspected SAP.

**Methods:**

Patients who underwent CAG for SAP were retrospectively screened. A total of 79 patients with no history of heart disease and wall motion abnormalities were included in the study. Forty-three patients with coronary lesions > 70% constituted the coronary artery disease (CAD) group and 36 patients without significant CAD constituted the control group. Layer-specific GLS transmural, endocardium, and epicardium values (GLS-trans, GLS-endo, and GLS-epi, respectively) were compared between the groups.

**Results:**

Patients in the CAD group had significantly lower GLS values in all layers (GLS-trans: -18.2 + 2.4% vs -22.2 + 2.2% *p* < .001; GLS-endo: -20.8 + 2.8% vs -25.3 + 2.6%, *p* < .001; GLS-epi: 15.9 + 2.4% vs -19.5 + 1.9%, *p* < .001). Multivariate adjustment demonstrated GLS-trans as the only independent predictor of CAD [OR:0.472, CI (0.326–0.684), *p* < .001]. Additionally, the GLS values were all lower in myocardial perfusion scintigraphy (MPS) true-positive patients compared with MPS false-positive patients (GLS-trans: -17.7 ± 2.4 vs. -21.9 ± 2.4%, *p* < .001; GLS-endo: -20.2 ± 2.9% vs -24.9 ± 2.9%, *P* < .001; GLS-epi: 15.4 ± 2.6% vs. -19.2 ± 1.8%, *P* < .001).

**Conclusion:**

Resting layer-specific strain as assessed by 2D speckle tracking analysis demonstrated that GLS values were reduced in all layers of myocardium with SAP and with no wall motion abnormalities. LSS analysis can improve the identification of patients with significant CAD but further prospective larger scale studies are needed to put forth the incremental value of LSS analysis over transmural GLS.

## Background

Coronary artery disease is one of the major causes of mortality and morbidity. Noninvasive imaging techniques (NIIT) are recommended in the diagnosis and risk stratification of patients with suspected stable angina pectoris (SAP) [1]. Resting transthoracic echocardiography is one of the leading tests used in the evaluation of patients with stable CAD. However, despite critical CAD, many patients do not exhibit wall motion abnormalities with resting conventional echocardiography when structural heart disease and history of prior myocardial infarction do not exist. In addition to TTE, exercise electrocardiography (ECG), myocardial perfusion scintigraphy (MPS), or stress echocardiography are widely used. Exercise ECG is the most widely available technique but has the lowest sensitivity and specificity. Nuclear imaging tests are chosen for providing high diagnostic accuracy but the major limitations are radiation exposure and lesser availability [2]. Dobutamine stress echocardiography is also a low cost and widely available technique without radiation exposure, it has a high sensitivity and specificity similar to nuclear perfusion scintigraphy, but the need of expertise limits its use [3]. Despite NIIT, nonsignificant coronary lesions are not a rare finding in patients undergoing elective coronary angiography. In a recently published study, the rate of significant CAD in elective coronary angiograms was 38% [4].

Myocardial strain analysis by two dimensional speckle tracking echocardiography (2D STE) has a higher diagnostic accuracy in detecting left ventricular dysfunction. Recently published studies revealed lower values of deformation in patients with acute coronary syndrome, diabetes mellitus [5], hypertension [1], and SAP [6–12]. In addition to global longitudinal strain (GLS), layer-specific strain (LSS) analysis provides the assessment of each myocardial layer separately. Strain analysis of longitudinal endocardial layer can give more accurate data about LV function and early signs of ischemia because endocardium is more susceptible to ischemia.

The aim of the present study was to evaluate layer-specific GLS in patients with suspected SAP and normal left ventricular wall motion.

## Methods

Patients who underwent diagnostic coronary angiography for SAP between January 2016 and January 2017 were screened from the electronic database. Patients with previous myocardial infarction, acute coronary syndrome, a history of coronary intervention or coronary artery bypass graft surgery, moderate-to-severe valvular disease, heart failure, segmental wall motion abnormalities, atrial fibrillation, reduced ejection fraction, and with malignancy were all excluded.

Echocardiographic images were assessed for suitability by an experienced cardiologist who was blinded to the other imaging results of the patients. All of the echocardiographic images were obtained using Vivid 7 Dimension or Vivid S70 systems (GE Healthcare, Horton, Norway) and imported to the EchoPAC workstation. Recordings with poor image quality that did not qualify for speckle tracking strain analysis were excluded. A total of 79 patients with good quality 2D echocardiographic images suitable for strain analysis, whose resting echocardiographic examination was performed within 1 week of the diagnostic coronary angiography were included in the study.

Conventional echocardiographic measurements were performed in accordance with the guidelines [13]. Biplane left ventricle ejection fraction was calculated using the modified Simpson method. End-systolic and end-diastolic diameters, and septal and posterior wall thickness were measured from the parasternal long axis view using M-mode. The diameter of the left atrium was measured using M-mode from the parasternal long view.

Two-dimensional speckle tracking strain analysis was performed by an experienced cardiologist according to the guidelines [14] from the recorded 2D grayscale images using Echopac software, without clinical knowledge of the patients. Cine-loop recorded, three beats of 2D images from 3 apical views (apical 2 chamber, 4 chamber and apical long axis views) with frame rates of 50–80 frames/s were accepted as suitable for strain analysis. In each view, regions of interests were outlined by defining one point on each side of the mitral annulus and one point at the apex. Then, the software automatically traced the borders of the LV myocardium; manual adjustments were made if necessary. Images with poor tracking quality and with more than one untrackable segments were excluded. After manual adjustments, the software calculated strain values in each view. End-systole was defined as aortic valve closure in the apical long axis view. A 17-segment bull’s eye view was formed after processing all three apical views. GLS-trans, endocardial, and epicardial values (GLS-trans, GLS-endo, and GLS-epi, respectively) were calculated automatically by the software. Regional longitudinal strain (RLS) was also calculated for all layers, based on the 17 segment model according to the perfusion territories of 3 major coronary arteries by averaging all segments peak strain values within each territory [15].

Intra-observer reliability was assessed by re-analyzing the images of 15 patients 30 days after the first analysis by the same operator. Inter-observer reliability assessment was performed by comparing the measurements of 15 randomly chosen patients, performed by another operator.

Coronary angiographic images were evaluated by an experienced cardiologist who was blinded to patients’ data. Lesions > 70% stenosis were accepted as critical stenosis. Patients with significant coronary artery disease were classified as the CAD group, and those without significant CAD were classified as the control group.

The study was conducted in accordance with Declaration of Helsinki and was approved by the local ethics committee.

### Statistical analysis

Statistical analysis was performed using IBM SPSS version 22 (IBM SPSS Statistics for Windows, Armonk, NY, IBM Corp). Categorical variables are expressed as numbers and percentages and continuous variables are expressed as mean ± standard deviation. The Kolmogorov-Smirnov test was used to assess the distribution of variables. Continuous variables were compared using the independent samples *t*-test or Mann-Whitney U test. Categorical values were compared using the Chi-square (χ^2^) test or Fisher’s exact test. The areas under the Receiver operating characteristics (ROC) curves and their area under the curve (AUC) were constructed for layer-specific GLS and RLS. Inter- and intra-observer reliability were evaluated using Bland-Altman analysis and intra-class correlation. A value of *P* < .05 was accepted as significant.

## Results

In total, 79 patients suspected of having stable coronary artery disease were included in the study; 36 without significant CAD (control group) and 43 patients with significant CAD (CAD group). The clinical data of the of the study population are given in Table 1. The ratio of female patients was higher in the control group [*n* = 24 (66.7%) vs. *n* = 11 (25.6%), *P* < .001]. There were no differences between the groups in terms of HT, DM, hyperlipidemia [16], and body mass index (BMI). Twelve (33.3%) of the patients in the control group had MPS, and 20 (46.5%) of the patients in the CAD group had MPS before CAG (*p* = 0.235). Most (83.4%) of the patients in the CAD group had LAD lesions.

**Table 1:** Clinical and angiographical characteristic of the patients

	CAD *n* = 43	Control *n* = 36	*P*
Demographic data and risk factors			
Age	60.4 ± 9.8	56.4 ± 8.1	.072
Male	32 (74.4%)	12 (33.3%)	<.001
Female	11 (25.6%)	24 (66.7%)	
HT	38 (88.4%)	32 (88.9%)	.943
DM	16 (37.2%)	10 (27, 8%)	.374
HL	30 (69.8%)	20 (55.6%)	.192
BMI, kg/m^2^	28.3 ± 5.0	29.7 ± 4.8	.252
MPS	20 (46.5%)	12 (33.3%)	.235
Coronary angiographic parameters			
One Vessel Disease	16	–	
Two Vessel Disease	14	–	
Three vessel disease	13	–	
LMCA	3	–	
LAD	36	–	
Cx	22	–	
RCA	25	–	

*BMI* Body-mass index, *CAD* Coronary artery disease, *Cx* Circumflex artery, *DM* Diabetes Mellitus, *HL* Hyperlipidemia, *HT* Hypertension, *LAD* Left anterior descending artery, *LMCA* Left main coronary artery, *MPS* Myocardial perfusion scintigraphy, *RCA* Right coronary Artery

Conventional echocardiographic measures and strain values are presented in Table 2. Layer-specific strain measurements were all lower in the CAD group (GLS-trans: − 18.2 ± 2.4% vs. -22.2 ± 2.2%, *P* < .001; GLS-endo: − 20.8 ± 2.8% vs. -25.3 ± 2.6%, *P* < .001; GLS-epi: − 15.9 ± 2.4% vs. -19.5 ± 1.9%, *P* < .001) (Table 2). A comparison of the difference between GLS-endo and GLS-epi revealed a lesser amount of difference in the CAD group (GLS-endo-epi, 5.0 ± 1.1 vs. 5.7 ± 1.2, *P* = .007). RLS values are given in Table 3, all layers in major coronary artery territories demonstrated significantly lower deformation values in patients with significant stenosis.

**Table 2:** Conventional echocardiographic parameters and longitudinal strain values

	CAD *n* = 43	Control *n* = 36	*P*
Echocardiogphic parameters			
LV EF, %	65.4 ± 5.3	66.4 ± 4.8	.426
LV EDD, mm	47.5 ± 4.8	47.9 ± 5.1	.737
LV ESD, mm	30.7 ± 4.8	30.9 ± 4.4	.933
LV mass, g/m^2^	98.7 ± 18.5	92.0 ± 22.5	.115
LA diameter, mm	38.7 ± 3.7	37.1 ± 2.9	.030
E/e’	8.7 ± 1.7	8.2 ± 1.4	.413
2D Global longitudinal strain (GLS) parameters			
GLS transmural, %	−18.2 ± 2.4	−22.2 ± 2.2	<.001
GLS endocardium, %	−20.8 ± 2.8	−25.3 ± 2.6	<.001
GLS epicardium, %	−15.9 ± 2.4	−19.5 ± 1.9	<.001
GLS endo-epi	5.0 ± 1.1	5.7 ± 1.2	.007

*CAD* Coronary artery disease, *EDD* End-diastolic diameter, *EF* Ejection fraction, *ESD* End-systolic diameter, *E* Pulsed wave transmitral early diastolic velocity, *e’* Early myocardial diastolic velocity, *GLS* Global longitudinal strain, *LA* Left atrium, *LV* Left ventricle

**Table 3:** Regional longitudinal strain values

	CAD	Control	*P*
LAD			
RLS transmural, %	−18.1 ± 2.4	−22.4 ± 2.7	<.001
RLS endocardium,%	−22.1 ± 3.3	−26.7 ± 3.5	<.001
RLS epicardium, %	−15.2 ± 2.4	− 19.2 ± 2.3	<.001
Cx			
RLS transmural, %	−16.6 ± 3.2	−21.2 ± 2.8	<.001
RLS endocardium,%	−18.8 ± 3.4	−24.2 ± 3.3	<.001
RLS epicardium, %	−14.8 ± 3.1	−19.0 ± 2.5	<.001
RCA			
RLS transmural, %	−19.2 ± 3.0	−22.7 ± 3.1	<.001
RLS endocardium,%	−21.3 ± 3.2	−25.0 ± 3.2	<.001
RLS epicardium, %	−17.7 ± 2.9	−20.8 ± 2.8	0.001

*CAD* Coronary artery disease, *Cx* Circumflex artery, *LAD* Left anterior descending artery, *RCA* Right coronary artery, *RLS* Regional longitudinal strain

**Fig. 1:**
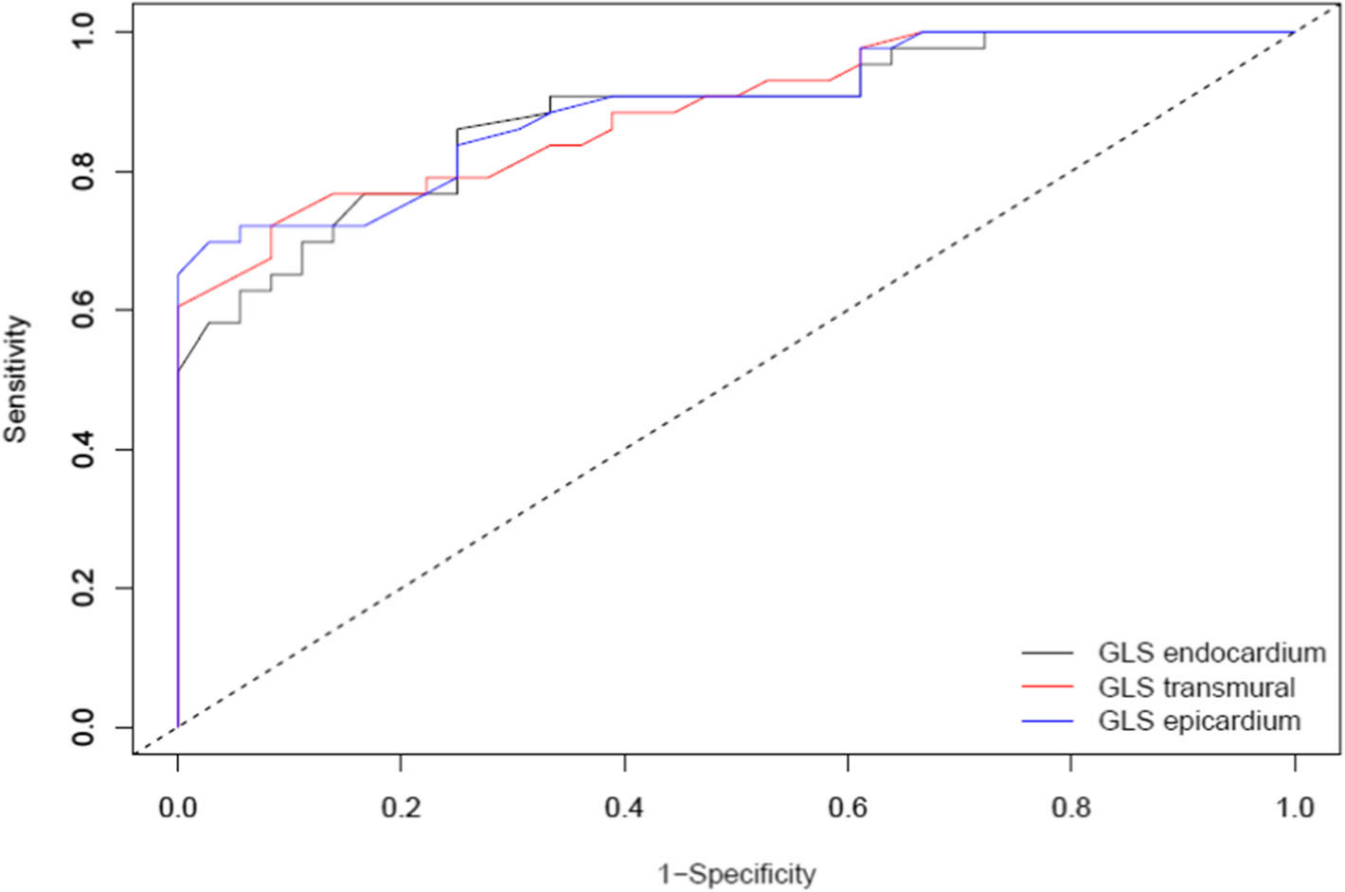
Receiver operating curves demonstrating value of layer-specific GLS for the diagnosis of CAD. Legends: GLS = Global longitudinal strain

**Fig. 2:**
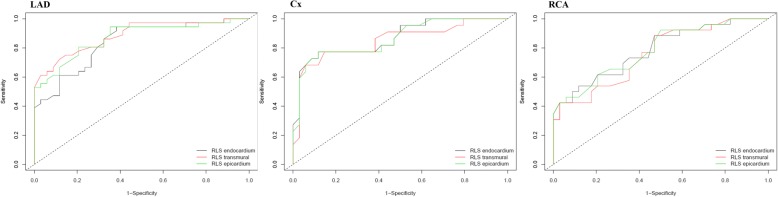
Receiver operating curves demonstrating diagnostic value of regional longitudinal strain for LAD, Cx and RCA. Legends: LAD: Left anterior descending artery, Cx: Circumflex artery, RCA: Right coronary artery, RLS: Regional longitudinal strain

ROC curves were constructed for layer-specific GLS in patients with CAD (Fig. 1). The diagnostic performance of GLS-trans, GLS-endo and GLS-epi were all significant. The cut-off values for GLS-trans, GLS-endo and GLS-epi were − 19.3%, − 23.4% and − 17.3%, respectively (Table 4). After multivariate adjustment (age, sex, BMI, HT, DM, GLS-endo, GLS-epi and GLS transmural) GLS-trans was found as the only independent predictor of CAD [OR:0.472, CI (0.326–0.684), *p* < .001]. ROC curves for layer-specific RLS were shown in Fig. 2 and the analysis of the curves with AUC were presented in Table 5. RLS_LAD_ and RLS_Cx_ had better predictive power for the detection of significant stenosis in the coronary artery supplying the associated territory, there were no differences between layers in terms of predictive value. (Table 5).

**Table 4:** Analysis of receiver operating characteristic curves and cut-off values for layer-specific global longitudinal strain

	AUC (95% CI)	Cut-off value	Sensitivity	Specifity	*P*-value
GLS transmural	0.891 (0.823–0.954)	−19.3%	69.8%	97.2%	<.001
GLS endocardium	0.881 (0.808–0.905)	−23.4%	86.0%	75.0%	<.001
GLS epicardium	0.885 (0.815–0.955)	− 17.3%	72.1%	91.7%	<.001

*GLS* Global longitudinal strain

**Table 5:** Analysis of receiver operating characteristic curves for layer-specific regional longitudinal strain

	AUC (95%CI)	*P*-value
RLS_LAD_ transmural	0.871 (0.787–0.954)	<.001
RLS_LAD_ endocardium	0.839 (0.746–0.931)	<.001
RLS_LAD_ epicardium	0.885 (0.808–0.962)	<.001
RLS_Cx_ transmural	0.862 (0.762–0.964)	<.001
RLS_Cx_ endocardium	0.866 (0.767–0.966)	<.001
RLS_Cx_ epicardium	0.848 (0.736–0.961)	<.001
RLS_RCA_ transmural	0.783 (0.667–0.899)	<.001
RLS_RCA_ endocardium	0.784 (0.667–0.900)	<.001
RLS_RCA_ epicardium	0.758 (0.636–0.880)	0.003

*Cx* Circumflex artery, *LAD* Left anterior descending artery, *RCA* Right coronary artery, *RLS* Regional longitudinal strain

Patients with MPS were grouped as MPS true-positive and MPS false-positive. A comparison of these two groups demonstrated lower strain values in all layers in the true- positive MPS group (GLS-trans: − 17.7 ± 2.4 vs. -21.9 ± 2.4%, *P* < .001; GLS-endo: − 20.2 ± 2.9% vs. -24.9 ± 2.9%, *P* < .001; GLS-epi: 15.4 ± 2.6% vs. -19.2 ± 1.8%, *P* < .001) (Table 6). Layer- specific strain analysis was compared between the sexes in both groups. There were no significant differences in terms of GLS between the sexes in both groups (Table 7).

**Table 6:** Layer specific GLS values in myocardial perfusion true positive vs false positive patients

Variable	MPS true positive *n* = 20	MPS false positive *n* = 12	*P*
GLS transmural, %	−17.7 ± 2.4	−21.9 ± 2.4	<.001
GLS endocardium, %	−20.2 ± 2.9	−24.9 ± 2.9	<.001
GLS epicardium, %	−15.4 ± 2.6	−19.2 ± 1.8	<.001

*GLS* Global longitudinal strain, *MPS* Myocardial perfusion scintigraphy

**Table 7:** Global lonigtudinal strain in female vs male patients

Control Group			
Variable	Female *n* = 24	Male *n* = 12	P
GLS transmural, %	− 22.5 ± 2.4	− 21.6 ± 1.8	.235
GLS endocardium, %	− 25.6 ± 2.8	−24.6 ± 2.1	.284
GLS epicardium, %	− 19.9 ± 2.0	− 19.1 ± 1.6	.250
Coronary Artery Disease Group			
Variable	Female *n* = 11	Male *n* = 32	P
GLS transmural, %	− 18.5 ± 2.6	− 18.1 ± 2.4	.664
GLS endocardium, %	− 21.0 ± 3.2	− 20.8 ± 2.8	.790
GLS epicardium, %	− 15.9 ± 2.9	− 15.9 ± 2.3	.949

*GLS* Global longitudinal strain

**Fig. 3:**
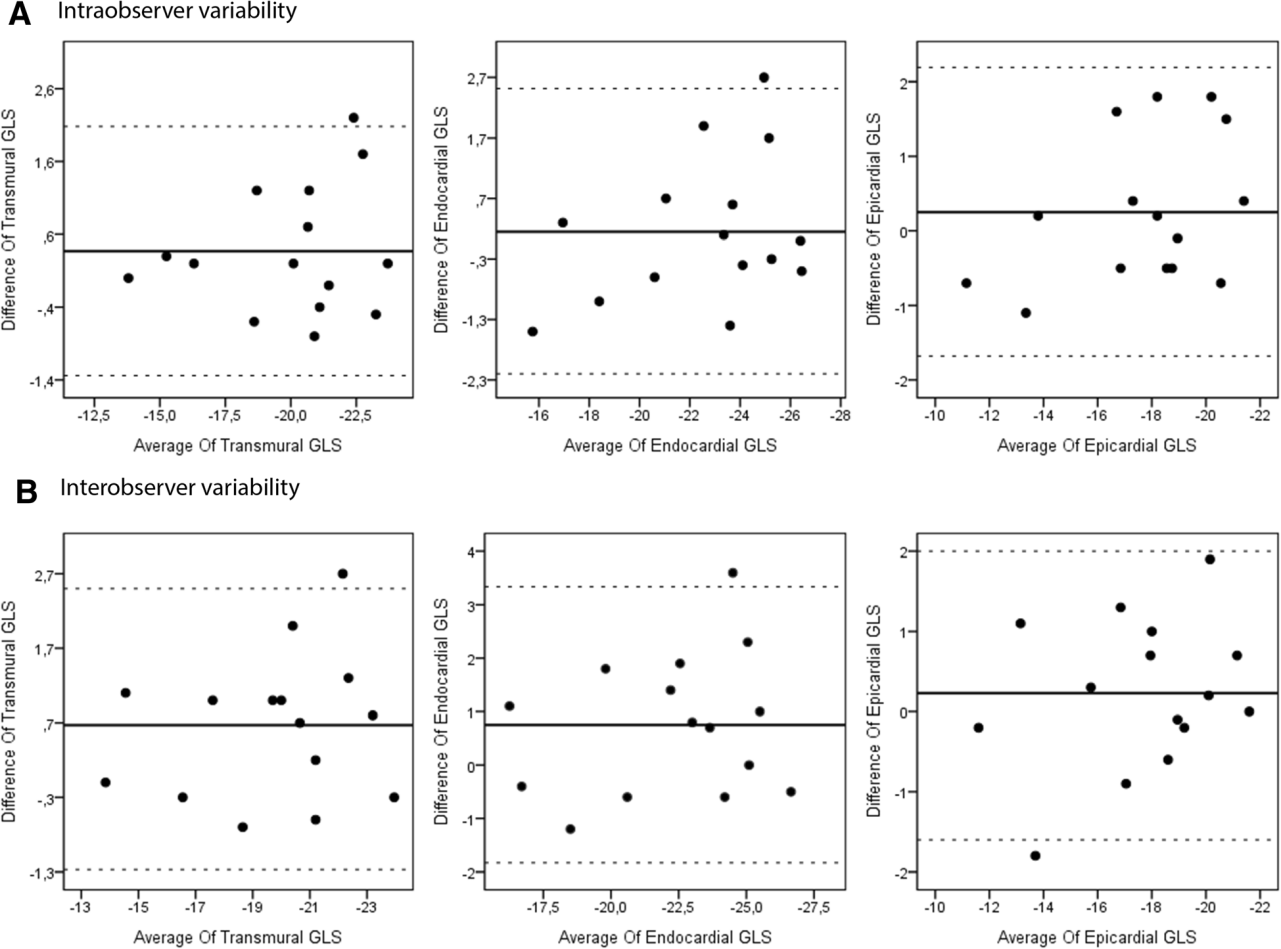
Intraobserver and interobserver variability analysis. Legends: Bland-Altman plots intraobserver (**a**) and interobserver (**b**) correlation for transmural, endocardial and epicardial longitudinal strain showing the mean difference and 95% limits of aggreement

Intra-class correlation and Bland-Altman analysis (Fig. 3) was used for the evaluation of intra- and inter-observer variability. Intra-observer reliability, as assessed by inter-class correlation coefficients for GLS-trans, GLS-endo, and GLS-epi were 0.957 (95% CI: 0.876–0.985), 0.937 (95% CI: 0.822–0.978), and 0.945 (95% CI: 0.844–0.990), respectively. Inter-observer reliability, as assessed by inter-class correlation coefficients for GLS-trans, GLS-endo, and GLS-epi were 0.950 (95% CI: 0.857–0.983), 0.920 (95% CI: 0.779–0.972), and 0.951 (95% CI: 0.860–0.983), respectively.

## Discussion

The current study demonstrated that GLS was significantly reduced in patients with stable CAD. LSS analysis revealed that all myocardial layers were affected in patients with significant CAD.

Evaluation of left ventricular function and wall motion analysis with conventional 2D echocardiography mostly fails to provide additional information, particularly when there is no history of prior myocardial infarction or structural heart disease. 2D speckle tracking strain analysis is a semi-automated technique that is more sensitive and accurate in measuring LV function than conventional 2D methods. Recently published studies demonstrated that longitudinal myocardial strain imaging with 2D speckle tracking had a diagnostic and prognostic value in patients with acute coronary syndromes [7, 17], and it has also been demonstrated as an independent predictor of significant CAD in patients with SAP [11]. Liou et al. [18] claimed that GLS could be an early marker of CAD in symptomatic patients. Stankovic et al. [5] demonstrated that strain imaging was superior to visual assessment in the detection of LAD stenosis. In line with these studies, we also demonstrated reduced transmural GLS in patients with significant CAD.

Although studies have been published showing the efficacy of GLS in detecting LV dysfunction with resting TTE examination [5, 12, 19], there are limited data about LSS imaging in stable CAD. LSS analysis allows us to assess each layer of the myocardium separately. Longitudinal endocardial strain is expected to be the most sensitive parameter for detecting significant CAD because ischemia initially affects the endocardium, indicating that LSS could provide additional information in patients suspected SAP. Multi-layer strain imaging has been assessed in patients with non-ST elevation acute coronary syndromes and the results indicated that it could be useful in identifying patients with significant CAD [6, 20]. The correlation of fractional flow reserve (FFR) values and regional LSS was investigated in a retrospective study in patients with SAP [21]. The results demonstrated lower transmural and endocardial longitudinal strain values in lesions with FFR < 0.75. A recently published study assessed LSS in patients with reversible ischemia using MPS [22]. In conclusion, the study revealed that all layers of the myocardium were affected in patients with significant CAD and claimed that both GLS and LSS could increase the diagnostic accuracy of single-photon emission computed tomography (SPECT) imaging. Similarly, in the present study we also investigated patients who underwent elective coronary angiography with suspected SAP. Our results were also in agreement with the study by Hagemann et al. [22], demonstrating that transmural, endocardial, and epicardial longitudinal strain (LS) values were all lower in patients with CAD compared with the control group. Additionally, in the current study, patients with true-positive and false-positive MPS were also compared and in line with the above-mentioned study, strain values were lower in all layers in patients with true-positive MPS (Table 6) [22]. Although it’s hard to draw a definite conclusion with this limited number of patients, these results also imply that 2D strain imaging might improve the diagnostic value of SPECT imaging, however, further prospective studies are needed.

Since endocardial thickening and shortening with systole is greater than epicardial changes, the deformation rate normally decreases from endocardium to epicardium [23–25]. In the current study, in parallel with the abovementioned studies, the gradient in strain values from endocardium to epicardium was apparent in both groups, whereas it was significantly lower in patients with significant CAD, indicating a higher reduction in the endocardial layer (Table 2).

In patients with CAD, ischemia extends from endocardium to epicardium. Initially subendocardial area is affected and endocardial LS deteriorates before epicardial LS abnormalities become apparent [21]. In line with other studies, the present study demonstrated that, in addition to endocardial and transmural LS, epicardial LS values had also reduced in CAD patients [6, 22, 26]. It should be noted that although the deformation among the layers of the myocardium is heterogeneous, it’s not independent from each other. Despite layered structure, the structural integrity of myocardium causes any deformation in one layer to affect the adjacent tissue. The deformation of one layer consists of active function of the layer and passive function from the adjacent layer. Furthermore, CAD severity and lack of collaterals, particularly in the presence of occluded coronary arteries, affect the extend of ischemia from endocardium to epicardium.

Although the ROC curve analysis demonstrated that transmural, endocardial and epicardial GLS had a value in the diagnosis of significant CAD (Fig. 1.), after multivariable regression analysis, only transmural GLS stayed independently associated with CAD. In contrast to the present study, endocardial layer was independently associated with CAD in the studies by Sarvari et al. [26] and Zhang et al. [6]. Additionally, as distinct from these studies, Hagemann et al. [22] claimed that epicardial and mid-myocardial GLS were better predictors of CAD.

Layer-specific RLS was also assessed in addition to GLS. Since CAD causes segmental wall motion abnormalities, RLS analysis sounds reasonable but there’s limited data. Liu et al. [20] demonstrated that endocardial GLS and RLS_LAD_ had higher accuracy in identifying LAD stenosis. In the current study, ROC curves for LAD, Cx and RCA territories demonstrated that transmural, endocardial and epicardial LS had diagnostic value but RLS_LAD_ and RLS_Cx_ had better discriminative power than RLS_RCA_ (Fig. 2) On the contrary, due to lack of segmental reference values, and higher inter-vendor variabilities, guidelines do not recommend strict RLS analysis [13, 27]. The mismatch between RLS and the specific territory of diseased coronary artery can be explained by the anatomical changes in the course of coronary arteries, and microvascular connections causing zones of dual arterial perfusion. Furthermore, it has also been shown that remote areas not supplied by stenotic coronary arteries had also lower strain values than control subjects [20].

Normal values for GLS and LSS have not been standardized yet. Inter-vendor variability, age and sex related changes in strain values are the main factors preventing the determination of cut-off value. It’s recommended to use the same software and vendor-specific normal reference values for interpretation [28, 29]. Marwick et al. [30] used the same vendor as we did and demonstrated an average GLS of − 18.6 ± 0.1%. Takidiki et al. assessed normal range of 2D LS and compared three vendors (− 21.3 ± 2.1% vs − 18.9 ± 2.5% vs − 19.9 ± 2.4, *p* < .001). Recently three studies, using the same vendor as we used, were published investigating the normal values of longitudinal LSS [23–25]. Nakata et al. [24] defined the normal values for transmural, endocardial and epicardial GLS as − 20.0 ± 2.0%, − 23.1 ± 2.3% and − 17.6 ± 1.9%, respectively, whereas the values found by Alcidi et al. for all layers were − 22.7 ± 1.8%, − 25.4 ± 2.1% and − 21.1 ± 1.8%, respectively. Shi et al. [23] also identified similar values for all layers (− 21.3 ± 2.9%, − 24.3 ± 3.1%, and − 18.9 ± 2.8%, respectively). In aggreement with these studies the cut-off values for GLS-trans, GLS-endo and GLS-epi were similar to the normal values abovementioned (− 19.3%, − 23.4% and − 17.3%, respectively).

There are numerous factors such as age, sex, DM, and HT that can affect longitudinal strain. Tadic et al. [31] demonstrated that patients with non-complicated DM and HT also had impaired LV longitudinal strain. In the present study, the rates of DM and HT were similar in both groups. However, the ratio of female patients was higher in the normal coronary angiography group, similar to previous studies. In the study by Sorenson et al. [11], the rate of male patients was 75% vs. 35% in patients with and without significant CAD, respectively. Previous studies have reported different results about the effect of sex on LSS parameters. Nakata et al. [24] and Shi et al. [23] demonstrated that female patients tended to have higher strain values compared with men, whereas Alcidi et al. [25] could not show a sex-specific difference. Although the results of the studies are conflicting, this difference can be attributed as a confounding factor. However, when we analyzed both groups separately in terms of sex and GLS, the results demonstrated that strain values did not differ between the sexes (Table 7).

### Limitations

The present study has several limitations. First, the retrospective nature of this study may have caused a loss of data and selection bias. Secondly, this is a small-scale study with a limited number of patients. As in all studies based on echocardiography, image quality and operator experience have a great effect on proper analysis. Randomized, prospective, multicenter, larger scale studies powerful enough to assess the effect of all confounding factors are needed to overcome these limitations.

## Conclusion

Resting layer-specific LS as assessed by 2D speckle tracking analysis demonstrated that GLS values were reduced in all layers of the myocardium in patients with SAP and with no wall motion abnormalities. These results indicate that GLS can improve the identification of patients with significant CAD but further studies are needed to put forth the incremental value of LSS analysis over transmural GLS.

## Abbreviations

2D STE: Two dimensional speckle tracking echocardiography
AUC: Area under the curve
CAD: Coronary artery disease
Cx: Circumflex artery
DM: Diabetes Mellitus
ECG: Electrocardiography
GLS: Global longitudinal strain
HT: Hypertension
LAD: Left anterior descending artery
LS: Longitudinal strain
LSS: Layer-specific strain
MPS: Myocardial perfusion scintigraphy
NIIT: Noninvasive imaging techniques
RCA: Right coronary artery
RLS: Regional longitudinal strain
ROC: Receiver operating characteristics
SAP: Stable angina pektoris
TTE: Transthoracic echocardiography
